# Hand grip strength as a proposed new vital sign of health: a narrative review of evidences

**DOI:** 10.1186/s41043-024-00500-y

**Published:** 2024-01-09

**Authors:** Raju Vaishya, Anoop Misra, Abhishek Vaish, Nicola Ursino, Riccardo D’Ambrosi

**Affiliations:** 1 Department of Orthopaedics, Indraprastha Apollo Hospitals, Sarita Vihar, New Delhi, 10076 India; 2 Department of Endocrinology, C-DOC Fortis Hospital, Nehru Place, New Delhi, India; 3 IRCCS Ospedale Galeazzi – Sant’Ambrogio, Milan, Italy; 4 Dipartimento di Scienze Biomediche per la Salute, Università Degli Studi di Milano, Milan, Italy

**Keywords:** Muscle, Sarcopenia, Frailty, Vital sign, Handgrip strength, Biomarker, Osteoporosis, Diabetes, Health

## Abstract

Hand grip strength (HGS) serves as a fundamental metric in assessing muscle function and overall physical capability and is particularly relevant to the ageing population. HGS holds an important connection to the concept of sarcopenia, which encompasses the age-related decline in muscle mass, strength, and function. It has also been reported to indicate the health of an individual. We reviewed the interplay between HGS and various health parameters, including morbidity and mortality, by carrying out a literature search on PubMed, Scopus and Google Scholar between 10 and 30 August 2023, to identify the relevant papers on the relationship between health and HGS. We used several keywords like ‘hand grip strength’, ‘muscle strength, ‘sarcopenia’, ‘osteosarcopenia’, ‘health biomarker’, ‘osteoporosis’, and ‘frailty’, to derive the appropriate literature for this review. This review has shown that the HGS can be measured reliably with a hand-held dynamometer. The cut-off values are different in various populations. It is lower in Asians, women, less educated and privileged, and those involved in sedentary work. Several diseases have shown a correlation with low HGS, e.g., Type 2 diabetes, cardiovascular disease, stroke, chronic kidney and liver disease, some cancers, sarcopenia and fragility fractures. The low HSG is also associated with increased hospitalization, nutritional status, overall mortality and quality of life. We believe that there is adequate evidence to show that HGS stands as an important biomarker of health. Its utility extends to the identification of diverse health issues and its potential as a new vital sign throughout the lifespan.

## Background and aims

Vital signs, comprising essential physiological markers like pulse, blood pressure, temperature, and respiratory rate, have traditionally formed initial medical evaluations. While these metrics offer a fundamental glimpse into patients' well-being, an evolving debate has spurred the consideration of other simple measurements. This expanded perspective encompasses parameters such as weight-related indices (body mass index, waist circumference), indicators of musculoskeletal strength (specifically handgrip strength, HGS), and the oxygen saturation state of the body (assessed through partial pressure of oxygen, pO2). Notably, the attractiveness of these measurements lies in their non-invasive nature, facilitating ease of implementation. Further, the advent of the COVID-19 pandemic has brought to the fore an escalating incidence of hyperglycaemia and prompted the introduction of blood glucose measurement as a new vital sign [1].

Importantly, HGS serves as a fundamental metric in assessing muscle function and overall physical capability. Particularly relevant to the ageing population, HGS holds an important connection to the concept of sarcopenia, which encompasses the age-related decline in muscle mass, strength, and function. While sarcopenia remains are fairly researched topic [2, 3], more prominently so during COVID-19 [4], HGS as an independent issue has received much less attention [5].

In this narrative review, we focus on the interplay between HGS and health parameters, including morbidity and mortality. Through a synthesis of empirical evidence, we propose HGS as a new vital sign in clinical practice.

## Methods

We carried out a literature search on PubMed, Scopus and Google Scholar between 10 and 30 August 2023, to identify the relevant papers on the relationship between health and HGS. We have used several keywords like ‘hand grip strength’, ‘muscle strength, ‘sarcopenia’, ‘osteosarcopenia’, ‘health biomarker’, ‘osteoporosis’, and ‘frailty’.

## Results and discussion

In the following discussion, we shall discuss measurements and fallacies of HGS, its determinants and associations with various diseases.

### Handgrip strength measurement

#### Test

The HGS is measured easily by a hand-held dynamometer (HHD), one popular example being Jamar’s hand-held hydraulic dynamometer (performancehealth.com/amfile/file/download/file/5061/product/67330/). Isokinetic dynamometers offer heightened precision over handheld dynamometers (HHDs) for measuring muscle strength, albeit with greater cost and bulkiness. HHDs and manual muscle testing (MMT) are commonly employed due to their practicality.

The HHD produces a measure of isometric strength that allows identifying not only muscle weakness of the upper limb but also providing an indicative of overall strength since it reflects the strength of the lower limbs [6]. If the HGS is tested in a standing position, it is likely to capture lower body and core muscle strength, which is used in balance and exertion of force. In contrast, the HGS tested in a seated position measures the strength of smaller muscle groups of the hand and wrist and is more localized to the upper body [7].

There are different ways of measuring HGS. A commonly followed procedure, based on protocols given below. Both the American Society of Hand Therapists (ASHT) protocol and Southampton University protocols have been used in various studies [8, 9] (Box 1).

**Box 1:** Procedure for performing handgrip strength [8–13]

Preparation: Ensure the participant is in a comfortable seated position with their feet flat on the floor and their arm resting on a flat surface. Some investigators have used other positions including standing, and found strength measurement to be similar to sitting position, while other have found higher HGS values in standing position [10]. A review of the measurement of grip strength in clinical and epidemiological studies: toward a standardized approach [11]	
Shoulder position: The shoulder should be in a neutral position, and the elbow should be bent at 90 degrees	
Wrist position: Wrist should be between 0 and 30° of dorsiflexion	
Calibration: Before starting, make sure the dynamometer is calibrated according to the manufacturer's instructions to ensure accurate measurements	
Grip Position: Instruct the participant to hold the dynamometer in their hand so that the handle rests comfortably against their palm. The handle should be adjusted for a snug fit, neither too tight nor too loose	
Grip Technique: The participant should be encouraged to grip the handle as firmly as possible without straining excessively. The four fingers should wrap around the handle, while the thumb rests on the other side. Ensure that the wrist is maintained in a neutral position	
Measurement: Ask the participant to squeeze the dynamometer with maximal effort for about 3 to 5 s. During this time, encourage them to exert their maximum strength. The participant should avoid any sudden jerks or movements during the squeeze	
Encouragement: “I want you to squeeze as hard as you can for as long as you can until I say stop”. (When the needle stops rising)	
Rest Between Trials: Allow a brief rest period (about 1 min) between each trial to prevent muscle fatigue that could affect subsequent measurements	
Repeat Measurements: Typically, three trials are conducted for each hand alternately. This provides a more accurate representation of the participant's handgrip strength. However, some investigators see one trial as equally reliable [12]	

Notably, the Jamar hydraulic dynamometer shows higher reliability within and between individuals [13]. Ensuring precise calibration significantly enhances reliability, necessitating regular recalibration. Alternate dynamometers like the mechanical Smedley and pneumatic Martin vigorimeter employ distinct mechanisms for HGS measurement. Concerning the Smedley dynamometer, it has shown excellent results regarding its laboratory-tested accuracy but, when applied among older adults, it did not produce comparable results to the Jamar hydraulic dynamometer. Limited agreement between Jamar and Takei dynamometers is observed. Conversely, a comparison between Jamar and Martin's vigorimeter in healthy older individuals reveals a strong correlation. Hydraulic dynamometers, Baseline and Saehan demonstrate validity, reliability, and comparability to the Jamar model [13].

##### Dominant HGS

Dominant HGS refers to the measurement of hand grip strength in the dominant hand, which is typically the hand someone uses for tasks that require precision or force. This measurement can provide insight into the specific strength of the hand that is more often used for activities like writing, eating, or lifting objects.

##### Relative HGS

Relative HGS takes into account an individual's body size or weight concerning their HGS. Relative HGS is computed as absolute HGS divided by BMI [14]. This measurement helps to account for differences in hand grip strength that might be influenced by an individual's overall size. Some studies pointed out that relative HGS might have an advantage in predicting the risk of cardiovascular biomarkers, metabolic profile, and other cardiometabolic disorders [15–17].

#### Normative values of HGS as evaluated in various countries

Normative values for HGS are available from various countries, and these depend on the ethnicity, gender, age and nutritional status of an individual. Specifically, reference values for HGS that fulfil these criteria are reported from the population of several countries, e.g., Great Britain [18], Australia [19], Canada [20], Korea [21], Germany [22], and Japan [23]. Since the reference values from various countries may differ caution must be taken in applying these to other populations [18, 19]. Some examples of country-specific HGS values are discussed ahead.

In 1994, Crosby et al*.* [24] studied the normal HGS and the difference between dominant and nondominant hands., in 214 volunteers using the Jamar dynamometer in a US population. The mean maximum grip for women was 81 lb. and for men was 137 lb., and only 60% of patients had maximum strengths at level 2. Furthermore, the majority of right-handed subjects had 10% stronger HGS in the dominant hand. Amaral et al*.* [25] in a population-based cross-sectional study of 1609 adults from Brazil recorded HGS values at maximum performance based on three measurements of the two hands. In general, men had the maximum HGS; 57% higher than women (43.4 kg vs. 27.6 kg), and also higher HGS levels in the different age groups. In both sexes, the highest HGS values were observed in the age group of 30–39 years (men, 46.9 kg; women, 29.4 kg), with a subsequent decline. Wang et al*.* [26] provided normative HGS values for older Filipinos using a Smedley spring-type dynamometer. Their results showed that men have significantly higher HGS than women and the dominant hand was stronger than the non-dominant hand, regardless of gender. Overall, HGS declined linearly with age, except in the 5th percentile non-dominant HGS of women, which exhibited a cubic relationship with age. This pattern suggests an acceleration of HGS decline among the oldest and most physically frail women. Pratt et al*.* [27] presented normative data and HGS thresholds to help identify those with, or at risk of low HGS in the Irish population (*n* = 9431). These authors reported a progressive decline in HGS from ~ 45 years of age. The National Health and Nutrition Examination Study (NHANES, USA, 2011–12) showed that the mean HGS was greater among men than women. It increased linearly for children and in a quadratic fashion among adults for both sexes. The HGS peaked in the 30- to 39-year age group for both men (216.4 lbs) and women (136.5 lbs) with subsequent age groups showing a gradual decline [28]. Bohannon et al*.* [29] reported a difference in some of the stratified HGS values from the NHANES and NIH Toolbox studies, from data from 13,918 participants. The authors conclude that due to the lack of conformity of the NHANES protocol with current recommendations, it is not recommended to use these values for broad application as reference norms [29].

Comprehensive data on population-based HGS values, or HGS values taken to compute sarcopenia data is lacking from South Asia. Pal et al. [30] enrolled 804 participants (mean age = 44.4 years) in north India. They found that the peak HGS was achieved in the 3rd/4th decades and the muscle strength/mass of Asian Indians was lower than that of Caucasians based on NHANES-III data. An HGS < 27.5 kg in males/ < 18.0 kg in females was defined as a cut-off to define sarcopenia in this sample. In a study of 1005 healthy adults, from Mumbai, western India, normative values of HGS were collected. The authors showed that men had significantly higher values of HGS at 0° of elbow flexion (37.8 kg) than women (22.12 kg). For the remaining positions of the elbow, the average HGS values were: 33 kg in men and 20 kg in women [31].

#### Cut-offs of hand grip strength: guidelines and selected studies

While some have provided cut points and percentiles for muscle weakness, the development of clinically meaningful ethnic-, sex-, and age-specific categories for muscle weakness from HGS measurements, similar to thresholds for BMI, would be more informative. Standardization of HGS assessments will help to reduce internal threats to validity, develop uniformity for HGS assessments, enable comparisons of results across studies that included measures of HGS, and move the application of HHD into clinical practice. At present, various guidelines include mostly those from high-income countries (HIC) and fewer from low and middle-income countries (LMIC). Dhar et al. [32] observed that although several sarcopenia guidelines are available, from the Western world and East Asia, these are not fully relevant for the South Asian population. Hence, they presented a consensus document (South Asian Working Action Group on SARCOpenia (SWAG-SARCO) that addresses the gaps in the current guidelines. It gives equal importance to muscle function, muscle strength, and muscle mass.

Table 1 lists European, American and Asian guidelines stating cutoff points for the HGS for males and females. Mostly these have been developed while defining definitions for sarcopenia. Asian guidelines demonstrate lower cutoff values than the European and American guidelines. The European Working Group on Sarcopenia in Older People (EWGSOP, the Sarcopenia Working Group) has a cuff point for low muscle strength at two standard deviations (SD) below the mean reference value, which is < 30 kg in men and < 20 kg in women [33], whereas in the Asian Working Group for Sarcopenia (AWGS) it was defined as < 26 kg in men and < 18 kg in women [34]. It is, however, to be noted that the evaluation methods of these two guidelines are different. EWGSOP group assessed the cut-off value of HGS from the healthy population age range. It used receiver operating curve analysis representing cutoff value to determine the walking ability of participants, whereas AWGS studied older people. A study on Chinese subjects of 2821 (1398 men and 1423 women) community-dwelling older people (≥ 60 years) and 409 (205 men and 204 women) young healthy adults (25–34 years) was done to evaluate the gait speed, HGS, and DEXA scan. The 28.5 kg for men and 18.6 kg for women were found as the cut-off for HGS [35].

**Table 1:** Hand grip strength cut-off points in various guidelines

Continent/country	Guidelines	Mean age	Cut-off for males	Cut-off for females
Europe	EWGSOP2 (Cruz-Jentof et al*.* 2019) [36]	77.0 years	< 27 kg	< 16 kg
USA	FNIH (Studenski et al., 2014) [37]	Male: 75.2 years, Female: 78.6 years	< 26 kg	< 16 kg
Asia	AWGS 2019 Consensus Update (Chen—LK et al., 2020) [38]	Not Available	< 28 kg	< 18 kg
India	Sarco-CUBES (Pal et al*.*, 2021) [30]	44.4 years	< 27.5 kg	< 18 kg

*AWGS* Asian Working Group for Sarcopenia *EWGSOP2* Revised European Working Group on Sarcopenia in Older People, *FNIH* Foundation for the National Institutes of Health; *Sarco-CUBES* Sarcopenia-Chandigarh Urban Bone Epidemiological Study

#### Factors influencing hip grip strength

##### Socio-demographic factors

a Ethnicity

Differences in body composition exist across ethnicities, as African Americans have greater muscle mass than Whites, Hispanic Americans, and Asians [39, 40], and the HGS is also better for Africans *vs.* others [41]. The HGS in South Asian men and women was ~ 5–6 kg lower than in the other ethnic groups (British Whites and British Africans), in the UK Biobank study [42]. In a survey from the Boston Area Community Health/Bone (BACH/Bone), 1,157 black, Hispanic, and white randomly selected Boston men ages 30–79 year was assessed, using dual-energy x-ray absorptiometry (DEXA), upper extremity strength by HGS, and the lower extremity physical function was derived from walk and chair stand tests. Racial/ethnic differences, with and without adjustment for covariates, were evident in all outcomes except for HGS [43]. In an analysis of the HELIUS study, the authors investigated if ethnic differences in HGS are a potential cause of ethnic disparities in T2D, in the studied six ethnic groups (2086 Dutch, 2216 South Asian Surinamese, 2084 African Surinamese, 1786 Ghanaian, 2223 Turkish and 2199 Moroccan origin participants). The authors reported that the HGS differed significantly across ethnic groups. After full adjustment, they found an inverse association of HGS with T2D that it did not differ substantially between ethnic groups, men and women, and lean and overweight individuals. They concluded that there was a large ethnic difference in the HGS and a consistent association of low HGS with prevalent T2D and suggested that the HGS may be investigated as a target for intervention or a marker to identify people at risk of T2D [44].

b Socioeconomic status

Arokiasamy et al*.* [45] reported that the wealth quintiles are positively associated with HGS among men across all countries, except in South Africa. The mean age-adjusted HGS ranged from 22.8 kg in Mexico to 40.9 kg in South Africa. Compared to all other countries, on average older men and women in India and Mexico had a lower HGS than the other studied population groups. Both older men and women in South Africa had higher HGS than older people in other countries. Type of work/job and nutritional status are also positively associated with HGS. Roland et al*.* [40] found that people of low socioeconomic status had lower HGS than those of higher socioeconomic status, in the Western Australian population. The Survey of Health, Ageing and Retirement in Europe (SHARE) study comprised 27, 351 participants aged 50 years or more from 11 countries. While the education and income effects were inconsistent in most countries, wealth consistently predicted HGS, in each country. After the adjustment for health measures, education, income and occupation effects disappeared, whereas log of wealth effects remained significant in both men and women. The authors concluded that the old-age socio-economic and financial circumstances (as measured by wealth) are associated with HGS, particularly among the least wealthy, while circumstances defined earlier in life as measured by education, income and occupation do not consistently predict HGS [46].

iii. Education and employment

Total education of a person of less than 6 years duration and income of less than 500,000 Korean won currency were associated with decreased HGS in older men in South Korea [47]. Unemployment and low education may be associated with decreased physical activity [48, 49]. Thus, providing resources and education that promote healthy behavior adherence may help preserve HGS at adult age.

##### Health behaviors

a Physical activity and fitness

Bann et al. [50] found that every 1-h increase in higher-light physical activity was associated with a 6-kg higher HGS among men, but not women in which population. And, a high sedentary time is responsible for sarcopenia due to physical inactivity. Kim et al*.* [51] studied 46,269 participants (aged ≥ 65 years), from South Korea, who participated in the 2019 National Fitness Award Project. Amongst them, 6.8% had the highest level of overall physical fitness, while 48.9% had the lowest level. The group with low HGS (< 28 kg for men and < 18 kg for women) had significantly higher odds of having the lowest level of overall physical fitness (odds ratio: 5.232 in men and 6.351 in women), after adjusting for age and body mass index. The authors concluded that HGS could estimate muscular strength and endurance, aerobic fitness, flexibility, balance skills, and coordination skills, as well as overall physical fitness level in older adults, and could be used as a substitute test for their physical fitness level in limited situations.

b Nutritional status

Lower HGS than the normative values are viewed as an overall indicator of malnutrition and its use is recommended for nutritional intervention [52]. HGS can independently predict nutrition status and change in nutrition status in hospitalized patients [53]. In medical inpatients at nutritional risk, HGS provides significant prognostic information about expected mortality and complication risks and helps to identify which patients benefit most from nutritional support [54]. Dietitians reported three areas in which the HGS provided value: assessing nutrition status, motivating patients, and monitoring responses to nutrition interventions. Specifically, they were able to shift away from focusing solely on the change in weight to focusing on functional ability and strength [55]. In Indian patients with cirrhosis, the HGS was found to be a reliable, reproducible, cost-effective tool to assess the nutritional status and outcomes of cirrhotics awaiting liver transplants [56].

iii. Abdominal obesity

There is a potential linkage between diminished HGS and abdominal obesity. Keevil's investigation [57] revealed an inverse relationship wherein each 10 cm augmentation in waist circumference correlated with a decrement of 3.56 kg in HGS among middle-aged and elderly males in Cambridge, UK. In a related context, Zamboni et al. [58] postulated that the age-associated escalation in overall adipose tissue and specifically abdominal adiposity exerts an adverse influence on pro-inflammatory markers. This, in turn, exacerbates the infiltration of adipose matter into skeletal muscle and myocytes (myosteatosis and intramyocellular lipid accumulation) [59], consequently compromising their structural integrity and force-generating capacity. Such fat deposition in muscles could be higher in some ethnic groups such as Hispanics [60] and Asian Indians [61, 62].

#### Associations of hand grip strength and morbidity and mortality

**Fig. 1:**
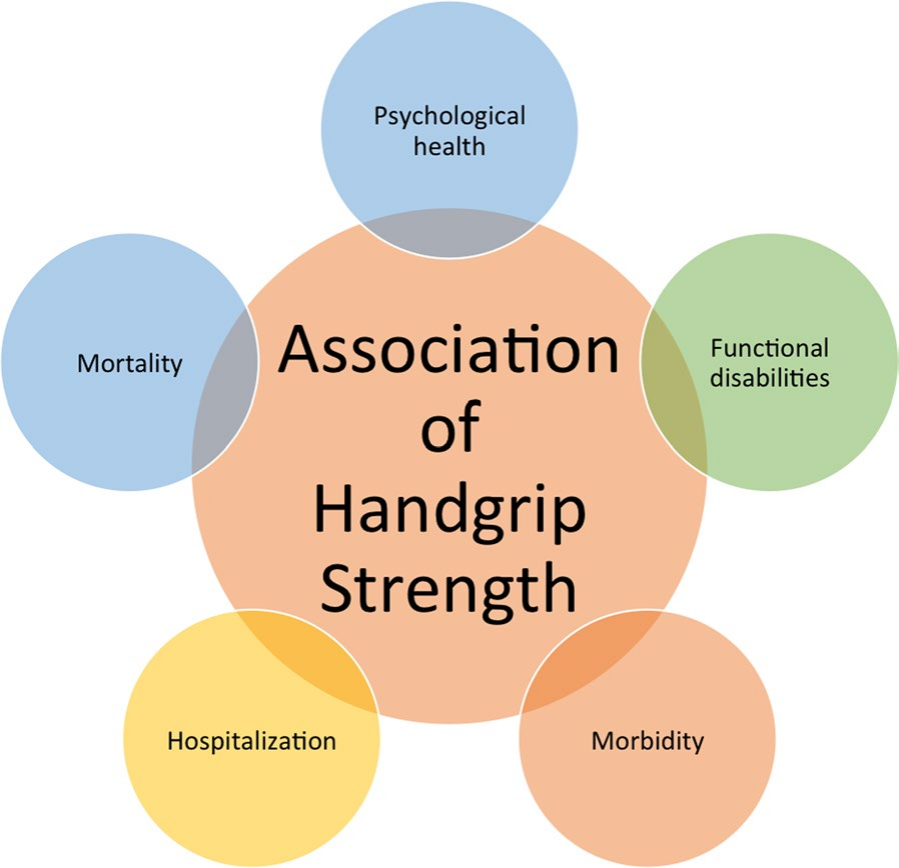
Associations of hand grip strength with health-related metrics

The reduction in muscle strength, as gauged by HGS can yield a spectrum of health consequences applicable to all population groups, although data are scarce from LMIC. Figure 1 depicts the association of HGS with various medical problems.

##### Metabolic syndrome and type 2 diabetes

Evidence from NHANES, USA (2011–2014) showed that the HGS was associated with insulin resistance and glucose metabolism in 959 adolescents (general, mixed population). Insulin and 2-h glucose levels decreased linearly as the HGS increased from the bottom quartile to the top quartile [63]. A study of adults from Hertfordshire, UK; aged 59–73 years showed that the HGS was associated with individual markers and summary definitions of metabolic syndrome [64].

In general, people with diabetes (almost all studies done in people with T2D) have low HGS [65] Data from several longitudinal cohort studies involving adults in the US, UK, and China have shown that low HGS is positively associated with risk of T2D [66, 67]. Data from the SHARE study, including 66,100 European participants aged 50 y or older free of diabetes at baseline that HGS was an independent predictor of new-onset diabetes. Moreover, relative HGS exhibited a slightly higher predictive ability than dominant HGS [68]. McGrath et al. [69] reported that those older Mexican Americans (*n* = 2270) who had a weaker normalized HGS at baseline had a higher risk for T2D compared to their stronger counterparts. Association between HGS and T2D prevalence in black, South Asian, and white European ethnic groups was done in a cross-sectional analysis of 418,656 participants (40–69 y) in the UK Biobank study. Lower HGS was associated with a higher prevalence of T2D, independent of confounding factors, across all ethnicities in both men and women. Additionally, the prevalence of T2D was three- to fourfold higher in South Asians and two- to threefold higher in black participants compared with white European participants across all levels of HGS. The authors concluded that the low HGS was associated with a disproportionately large number of T2D cases in South-Asian men and women and black men [42].

In the Korean population, lower relative HGS was associated with a higher risk of not only T2D but also impaired fasting glucose in both sexes. These trends were stronger in younger adults than in older adults [70]. However, not all studies show this association. The Prospective Urban–Rural Epidemiology (PURE) study reported that there was no association between HGS and the incidence of diabetes after surveying 17 countries with different income levels [71].

Low HGS often signifies compromised muscle quality, potentially instigating metabolic dysregulation and insulin resistance as individuals age [72]. This interplay might involve reduced skeletal muscle mass, impaired mitochondrial function, and altered adipokine secretion, collectively contributing to metabolic disturbances and insulin sensitivity decline. The presence of chronic diseases such as T2D is especially problematic for potentiating the disabling process in older adults because those with low HGS, and diabetes-associated peripheral neuropathy rapidly reduce muscle strength (“neurosarcopenia”).

##### Cardiovascular disease and mortality, and all-cause mortality

A Muscle strength is correlated with several vascular and cardiac pathologies:

(A) *Arterial Stiffness*: Evidence indicates that the release of myokines from skeletal muscle preserves or augments vascular function [73] and low HGS has been correlated with high arterial stiffness [74].
(B) *Biomarkers of CVD*: The associations between reduced HGS and biomarkers of chronic disease (e.g., hypertension, dyslipidaemia, etc.) may explain why low HGS is associated with CVD. A longitudinal study of Finnish adults (*n* = 963), with a follow-up of 22 years, reported that a decline in HGS was associated with CVD and hypertension [75]. Lawman et al. [7] in 4,221 US adults found a significant association between higher relative muscular strength, HGS and more favourable CVD biomarkers like systolic blood pressure, HDL cholesterol, triglycerides, and plasma insulin and glucose.
(C) *Cardiovascular Disease (CVD)*: In 2427 Korean adults, every increase of 1 in HGS reduced the 10-year CVD risk by 1.76 times (95% CI 1.58–3.71, *p* < 0.001), and when the waist-to-height ratio was < 0.50, the CVD risk decreased by 3.3 times (95% CI 0.16–0.56, *p* < 0.001) [76–81]. In South Korean people with T2D (*n* = 1250), The combination of low skeletal muscle mass and HGS was strongly associated with CVD, coronary heart disease, and peripheral artery disease, especially in those with higher HbA1c levels [82]. In an Austrian study of 691 individuals followed up for 9.2 + 3.1 y, HGS predicted major and total cardiovascular events [83].
(D) *Stroke*: In a longitudinal study of three prospective cohorts, the data from the Health and Retirement Study (HRS), the SHARE Study, and the China Health and Retirement Longitudinal Study (CHARLS) was used. A total of 4407 participants suffered from stroke and 9509 from heart disease during follow-up. Compared with the highest quartile, participants in the lowest quartile of dominant HGS, absolute HGS and relative HGS possessed a significantly higher risk of new-onset stroke in Europe, America, and China [84].
(E) *Congenital Heart Disease*: It has even been used as a diagnostic and prognostic marker in congenital heart disease [85].

b Mortality

Overall, the rank order for the top 10 risk factors (Table 2) for major CVD and all-cause mortality across the globe shows that population attributable fraction (PAF) for CVD, HGS ranked 9th, just below diabetes, while for PAF) of CVD mortality, it is ranked at the 3rd place [86].

**Table 2:** List of top 10 risk factors for major cardiovascular disease and all-cause mortality globally

Rank	CVD overall PAF (95% CI)	Mortality overall PAF (95% CI)
1	Hypertension: 22.3 (17.4–27.2)	Low Education: 12.5 (10.7–14.3)
2	High non-HDL Cholesterol: 8.1 (3.1–31.2)	Tobacco Use: 11.3 (8.1–14.5)
3	Household air pollution: 6.1 (4.5–7.6)	Low Hand Grip Strength: 11.6 (7.3–16.0)
4	Tobacco use: 6.1 (4.5–7.6)	Poor diet: 11.1 (7.7–14.6)
5	Poor diet: 6.1 (2.8–8.8)	Hypertension: 8.8 (7.6–9.9)
6	Low education: 5.8 (2.8–8.8)	Household air pollution: 6.6 (4.7–8.5)
7	Abdominal obesity: 5.7 (1.7–9.8)	Diabetes: 5.5 (4.2–6.8)
8	Diabetes: 5.2 (2.9–7.4)	Abdominal obesity: 2.8 (1.3–4.3)
9	Low hand grip strength: 3.3 (0.9–5.7)	Depression: 2.2 (1.4–3.0)
10	Low physical activity: 1.5 (0.3–2.7)	Low physical activity: 2.2 (1.0–3.3)

*CVD* cardiovascular disease, *PFA* population attributable fraction, *CI* confidence interval, Adapted from Lopez-Jaramillo et al. [86]

Wu et al. [87] evaluated 42 studies with 3,002,203 participants, in a Systematic Review.. For the lowest *vs*. the highest category of HGS, the hazard ratios (HRs) were 1.41 for all-cause mortality, 1.63 for CVD and 0.89 for cancer. The HRs with a per-5-kg decrease in HGS were 1.16 for all-cause mortality, 1.21 for CVD, 1.09 for stroke, 1.07 for coronary heart disease (CAD), and 1.01 for cancer. The authors concluded that the HGS was an independent predictor of all-cause mortality and CVD in community-dwelling populations.

A study of middle-aged and older German adults found men and women in the highest HGS quintile at baseline had, respectively, a 32% and 25% lower risk for all-cause mortality compared to those in the lowest HGS quintile. The older adults in the lowest HGS tertile had a 3.33 higher hazard ratio for all-cause mortality compared to persons in the highest HGS tertile [88]. Further, in the PURE study (*n* = 139,691) during a median follow-up of 4.0 years, the HGS was inversely associated with all-cause mortality. The study results showed that every 5-kg decrease in HGS was associated with a 16% higher HR for all-cause mortality. Further, HGS was found to be a stronger predictor of all-cause and CV mortality than systolic blood pressure in this study. It is pertinent to note here, that, as discussed previously, there was no association seen in HGS and T2D in this study [65]. In a representative sample of adults aged 50 years or older, data from 29 countries including 121,116 participants (276,994 observations; mean age 63.7 years; 56.3% women) free from prior heart attack or stroke were evaluated. An increase of 5 kg in HGS was associated with a reduced risk of all-cause [HR 0.86, 95% confidence interval (CI), 0.86–0.90], overall cardiovascular (HR 0.86, 95% CI 0.82–0.86), heart attack (HR 0.90, 95% CI 0.86–0.95), and stroke (HR 0.86, 95% CI 0.82–0.90) mortality [89]. Wei et al. [90] in a recently published study on 13,392 Chinese participants with T2D, found 3006 (22.45%) deaths, including 746 (5.57%) CVD deaths. The risk for all-cause mortality and CVD mortality among both men and women increased progressively with decreasing HGS quartiles. The authors concluded that the HGS displayed a linear downward trend with mortality risk among people with T2D patients, whereas muscle mass showed a U-shaped relationship. In addition, they commented that the low HGS seemed to be a better predictor for mortality compared to low muscle mass [90].

##### Sarcopenia, osteoporosis and frailty

(A) *Sarcopenia, Frailty and Hip Fractures*: Although Sarcopenia is a common clinical condition, prevalent amongst all global societies, its awareness and impacts are lesser known among clinicians and researchers, especially in lower-middle-income countries, as evidenced by the lesser number of publications so far on this subject [2]. It is well-recognized that hip fractures affect older individuals (> 65 years) [91, 92], due to the coexistence of osteoporosis and sarcopenia, which is also known as ‘osteosarcopenia’ [93]. Falls in older individuals are quite common and are the leading cause of hip fractures [94, 95]. Fragility fractures, especially of the hip gained importance and attention during the COVID-19 pandemic and posed several challenges in timely diagnosis and adequate management [96]. Frailty is associated with chronological age [97], but Syddall et al. [98] demonstrated a stronger association between frailty and (decreased) age and gender-stratified HGS. These authors suggested that HGS could be used as a single marker for frailty. Several instruments and scores for diagnosing and monitoring frailty have been developed, and many of them include HGS measures [99100], an example being the currently most cited score, Fried’s Phenotype [101]. It includes HGS as one of two objective measures, together with gait speed, the same criteria as used for diagnosing sarcopenia [33, 34]. Denk et al. [102] in a systematic review of 21,197 patients including 1392 hip fractures found an association between decreased HGS and low-impact hip fractures. However, these authors were not able to quantify the strength of this relationship, due to the heterogeneity of the included studies. The authors suggested that the HGS measurements may be useful for identifying individuals who might be at elevated risk of hip fracture. In this respect, it is pertinent to note that sarcopenia and frailty have increased after the COVID-19 pandemic, and may form a part of “long COVID (post-COVID-19) syndrome” [4]. It is useful to evaluate this by HGS measurements since low HGS has been seen along with fatigue in such patients [4].
(B) *Functional Disabilities*: Age-related sarcopenia elevates the risk for functional disabilities. It is known that lower HGS is associated with impairments in instrumental activities of daily living (IADL) and activities of daily living (ADL) [69, 103–105] as compared to those with higher HGS. A 25-year prospective cohort study, on Japanese-American men living in Hawaii, studied 3218 survivors. The risk of self-care disability was more than twice in the lowest *vs.* the highest grip strength tertile. They concluded that among healthy 45–68-year-old men, HGS was highly predictive of functional limitations and disability 25 years later. Good muscle strength in midlife may protect people from old age disability by providing a greater safety margin above the threshold of disability [106].

##### Other diseases

(A) Chronic Kidney Disease (CKD)
  The CKD is associated with impaired muscle strength both in adults and in children [107]. Reduced values of HGS are often found in patients undergoing dialysis therapy for end-stage renal disease [108]. An Indian study on 83 patients showed that there was a significant association with serum creatinine and HGS [109].
(B) Chronic Liver Disease (CLD)
  HGS was found inversely associated with the risk of NAFLD but also with its severity. [110]. In a Japanese study, Nishikawa et al. [111] found that liver fibrosis markers (like M2BPGi) were well correlated with HGS in CLD patients. In another study, Yoh et al. [112] concluded that the HGS can be an independent predictor for composite hepatic event development in patients with CLDs. Sex-specific risk of mortality in cirrhotic patients was increased in those with low HGS, regardless of coexistent hepatocellular carcinoma and the Child-Pugh class [113].
(C) Cancers
  Eight cancer sites were found to be inversely associated with HGS, namely, endometrium, liver, gallbladder, kidney, oesophagus, pancreas, colorectal, breast, and all-cause cancer in the UK population (*n* = 445,552). It is noted that the head, neck and breast cancers might be better predicted by relative grip strength [114]. Higher muscle strength at the start of palliative chemotherapy is associated with significantly better overall survival in older patients with advanced cancer [115]. Consistently, lower muscle strength was measured in breast cancer patients as compared to healthy women. In shoulder and knee strength in patients after chemotherapy. On average, patients had up to 25% lower strength in their lower extremities and 12–16% in their upper extremities, during cancer treatment compared with healthy women [116].
(D) Chronic Respiratory Diseases
  Jeong et al. [117] did not find any significant difference between the HGS of the participants with and without Chronic Obstructive Pulmonary Disease (COPD). However, low HGS was associated with decreased Quality of Life—including mobility, daily activity, pain/discomfort, and anxiety/depression—in patients with COPD.

Box 2 summarizes the associations of HGS and various factors and parameters of health.

**Box 2:** Handgrip Strength and Association with diseases and health-related problems

Morbidity	
A) Non-communicable diseases	
Type 2 diabetes	
Metabolic syndrome	
Cardiovascular diseases	
Dyslipidaemia	
Hypertension	
Cancers	
Non-alcoholic fatty liver disease	
Chronic liver disease	
Chronic kidney disease	
Chronic respiratory diseases	
Cognitive dysfunction and impaired mental health	
B) Musculoskeletal problems	
Chronic low back pain	
Osteosarcopenia	
Osteoporotic fractures	
Mortality	
All-cause mortality	
Cardiovascular diseases-related mortality	
Cancer-related mortality	
In-hospital mortality	
Post-operative mortality	
Cirrhosis-related mortality	
Other health-related problems	
Nutritional status	
Institutional admissions	
Longer hospital stay	
Reduced quality of life	
Functional disability	

##### Institutional admissions

Low HGS is identified as an important factor in older adults that is associated with higher nursing home placements or hospitalizations [118, 119]. This is of concern because those older adults who died in hospitals had longer hospital stays than all the patients [120].

##### Psychological Health

Lower HGS than the normative values are known to be linked with various psychological problems like cognitive impairments, poor mental health, depression etc.

- Cognitive impairments and poor mental health

Cognitive decline and HGS are correlated based on various studies. Boyle et al*.* [121] have demonstrated a bidirectional relationship: cognitive impairment is observed to be linked with diminished HGS, while lower HGS levels are concurrently associated with cognitive decline. Further, in a cross-sectional study of older men and women, those in the highest HGS quartile, had 62% and 49% lower odds, respectively, for cognitive impairments compared to those in the lowest HGS quartile [122]. In addition, a study revealed each 1-lb annual decline in HGS was associated with a 9% increased risk for Alzheimer’s disease [123].

In a study, more insight into the development of HGS across ages in people with intellectual disabilities (ID) was done, which was also compared with the general population. The authors demonstrated that people with ID have very low levels of HGS during their entire life [124].

b. Depression

Fukumori et al. [125] studied 4314 Japanese subjects from community-dwelling individuals aged 40–79 years and reported that the subjects with lower HGS (per 1SD decrease) had higher odds of having depressive symptoms at baseline. Further, lower HGS was associated with the longitudinal development of depressive symptoms after 1 year.

##### Quality of Life

In a cross-sectional study, the correlation of HGS (measured using a Jamar dynamometer) with the quality of life (measured using the EQ-5D and EQ-5D VAS questionnaires) of 123 elderly Indonesian patients was done. The HGS showed a significant correlation with the quality of life of elderly patients as measured using two questionnaires [126]. In an Austrian study on 63 older participants (60–94 years), social contacts with non-relatives and HGS, in contrast, had a significant positive impact on health-related quality of life among old-aged men and women. Physical well-being and in particular muscle strength estimated by HGS may increase health-related quality of life. [127].

The main findings of this review are listed in Box 3.

**Box 3:** Hand grip strength and health: summary of evidence

Measured reliably with a hand-held dynamometer	
HGS cut off points are different in various populations	
Lower values are seen in:	
Asians (than Caucasians and Africans)	
Women (than Men)	
Less dominant hand	
Less educated people	
Less privileged/wealthy	
Sedentary activities	
Malnutrition	
Abdominal obesity	
Associated with various diseases, hospitalizations, mortality, functional disabilities, and nutrition (See Box 2)	

### Hand grip strength as a clinical vital sign of health

The above discussion has shown that the HGS shows notable consistency in explaining diverse parameters including concurrent overall strength, upper limb function, bone mineral density, fracture susceptibility, T2D, CVD, mortality, nutritional deficiency, cognitive decline, depression, cancer and overall quality of life. Consequently, advocating the routine adoption of HGS is viable, whether as an independent metric or as an integral facet of a concise measurement set, to effectively identify older adults at risk of suboptimal health status.

Although the HGS has been indicated as an important and reliable biomarker of health, it is still not considered a vital sign. We believe that HGS should be a vital sign as it has the following unique features, which are crucial for the health of an individual:

(A) *Comprehensive Health Assessment*: While traditional vital signs offer insights into cardiovascular and respiratory well-being, HGS introduces a novel dimension by directly assessing musculoskeletal, and overall strength. This is crucial as it provides a more holistic perspective on an individual's physical condition.
(B) *Early Disease Detection*: Research consistently demonstrates that low HGS is associated with various chronic diseases, as stated above. By including HGS as a vital sign, healthcare practitioners can identify potential health issues at an earlier stage, enabling timely interventions and improved patient outcomes.
(C) *Predictive Value*: HGS has exhibited predictive capabilities for adverse health outcomes. Studies have shown that individuals with low HGS are at a higher risk of disability, hospitalization, and mortality. Incorporating HGS into routine health assessments could enhance risk stratification and preventive strategies.
(D) *Serial Measurements*: Simply performed serial measurements could indicate the progress of skeletal muscle and overall strength and patient engagement (see below).
(E) *Patient Engagement*: The simplicity of HGS measurement encourages patient engagement in their health management. By tracking their handgrip strength over time, individuals can monitor changes in their physical well-being, motivating them to adopt healthier lifestyles and adhere to medical recommendations.
(F) *Cost-Effective and Non-Invasive*: Measuring HGS is non-invasive, cost-effective, and requires minimal equipment. This aligns with the practicality of existing vital sign assessments, making it feasible for integration into routine clinical practice without imposing significant burdens on healthcare systems.
(G) *Diverse Applications*: HGS transcends age and gender, making it applicable across diverse populations. From assessing the health of ageing individuals to monitoring physical progress in rehabilitation settings, its versatility underscores its potential as a universally relevant vital sign.

## Study limitations and strengths

We acknowledge the following limitations of this review on this topic:

1. Normative HGS values vary across countries due to differences in ethnicity, gender, age, and nutrition, making it challenging to apply reference values to diverse populations. This lack of standardized values hinders accurate assessments.
2. Lack of data in many countries: Comprehensive population-based HGS data from several countries (e.g., South Asia) is limited, making it challenging to understand the health implications and norms in this region.
3. Paucity of standardized guidelines: The absence of globally standardized guidelines for HGS cutoff points for conditions like sarcopenia and other health parameters limits the consistency and comparability of results across studies.
4. Confounding factors and associations: While HGS is associated with various health conditions, it is often influenced by numerous confounding factors, making it challenging to establish causation and accurately predict health risks. This is increasingly challenging given paucity of data in several countries and populations.

The following are strengths of our review:

We believe that available data, and detailed review of various parameters carried out by us, are robust enough to show good association of HGS to various health parameters and mortality. Such detailed review on this topic brings novel and fresh perspective underscoring need to propose HGS as a new vital sign.

## Future directions

We are optimistic that the utility of HGS would extend to the identification of diverse health issues in the elderly population and its potential to complement other vital sign measurements throughout the lifespan. This highlights its promise for early intervention strategies aimed at disease prevention and health promotion. However, future research should prioritize the establishment of standardized protocols for HGS assessments to enhance their clinical applicability and accuracy.

## Conclusion

Several diseases have shown a correlation with low HGS, e.g., Type 2 diabetes, cardiovascular disease, stroke, chronic kidney and liver disease, some cancers, sarcopenia and fragility fractures. The low HSG is also associated with increased hospitalization, nutritional status, overall mortality and quality of life. The thorough analysis of existing data and various health parameters demonstrates significant associations between HGS and health outcomes, including mortality. This study suggests that HGS could be proposed as a new vital sign, offering valuable insights for clinical practice and public health.

## Data Availability

Data related to this study is available with the corresponding author if needed.

## Abbreviations

HSG: Hand grip strength
CVD: Cardiovascular disease
COVID: Coronavirus disease;
HHD: Hand-held dynamometer
MMRT: Manual muscle testing
T2D: Type-2 diabetes
CKD: Chronic kidney disease
CLD: Chronic liver disease
